# Observations on Functional Disorders of the Spinal Cord, and Their Connexion with Hysteric, Nervous, and Other Diseases; Illustrated by Cases, Selected Chiefly from the Reports of the Pallas-Kenry and Currah Dispensaries

**Published:** 1830-01

**Authors:** Wm. Griffin, D. Griffin

**Affiliations:** Limerick.; Limerick.


### FUNCTIONAL DISORDERS OF THE SPINAL CORD.

*Observations on Functional Disorders of the Spinal Cord, and their Connexion with Hysteric, Nervous, and other Diseases; illustrated by Cases, selected chiefly from the Reports of the Pallas-Kenry and Currah Dispensaries.* By Wm. Griffin, M.D. and D. Griffin, M.R.C.S. Limerick.

(Continued from the preceding vol. page 489.)

II. The case of Mrs. H., aged forty-five years, which occurred about the same time, was equally strange and interesting. She was seized with acute pain and great tenderness in the direction of the ascending branch of the colon, with costiveness, thirst, heat of skin, hard, quick pulse, and the most incessant vomiting. In a few hours the tenderness became so great, she could scarcely bear the slightest touch; and the irritability of stomach so extreme, that even the smallest quantity of fluid was instantly rejected. Bloodletting, mild purgatives, and eventual blistering, gave considerable relief; but the sickness of stomach continued, with little or no intermission: saline draughts, opium, all the usual remedies, failed to allay it.

The remission which had taken place in the other symptoms lasted only for two or three days; at the termination of which they recurred in their original violence, but the pain now occupied the situation of the transverse arch of the colon. There was much general fever, with flushing and costiveness as before, and relief was again obtained by a repetition of the bleeding, purging, and blistering. The attack, however, occurred a third time in the sigmoid flexure; and the bleeding, &c. was once more resorted to.

Though every accession of this complaint exhibited strong characters of acute inflammation, it did not, either in its disposition to resolve or to run its course to a fatal event, bear any resemblance to it. The same symptoms returned again and again, under a variety of treatment, but always attacking in succession new portions of the alimentary canal, at intervals of three or four days. The distressing vomiting continued throughout.

After several weeks had elapsed, without any material amendment, except that there was less general fever, and hardness of pulse, and more of debility and emaciation, a consultation was held. There was found, on examination, some apparent hardness of liver, and it was thought slight enlargement; and, as the evacuations had always been scanty, and of a dark colour, intermixed sometimes with green leafy bile, the symptoms were supposed to depend on a deranged state of that organ. A mercurial course was, in consequence, agreed on: copious ptyalism was induced, and was followed by a slow but progressive amendment. The vomiting abated; the frequent pain gradually wore away; and, though the lady remained for a long time in an enfeebled and emaciated condition, and was for months subject to slight returns of the complaint, she eventually attained a tolerably good state of health.

The resemblance which the first of these singular cases bears to some of Dr. Monteith's, quoted in Dr. Abercrombie's work, must at once occur to the reader. It seems an exact counterpart in many of the symptoms, the headach, palpitations, cataleptic fits, violent spasmodic affections of the abdomen, incessant vomiting, and in the impairment of vision. As it will be hereafter shown that spinal tenderness almost invariably exists at the very commencement of these attacks, it seems inexplicable that no such symptom should have existed in Dr. Monteith's cases. He states that no disease could be discovered on examination, or by pressure, though in some pain was increased by motion or attempting the sitting posture. It may possibly be, that the examination was not made with sufficient minuteness, as cases occur occasionally in which the tenderness is not at first apparent, and yet, on detecting it, it is found to be very acute. Though no examination was made in the early stage of the first complaint, of which the above history is given, and none at any period of the second, there is not the slightest doubt, from inferences fairly deduced in subsequent inquiries, that in both it might have been detected on the first day of attack, and the true nature of the disease at once ascertained.

The case of the younger lady was highly remarkable in its perfect imitation of almost every possible form of organic visceral disease, in the wonderful endurance of the system under such a continuance of deep and excruciating suffering, and in the apparent existing possibility of recovery after long years, during which every function of the constitution had been successively interrupted or disturbed, and every hour had been the harbinger of some new pain. Whether, in a case so intense, if treatment had been earlier directed to the spine, the disease would have been simplified and rendered more tractable, is questionable; but it, at all events, furnishes ample proof of the fruitlessness of all modes of cure merely directed to symptoms.

The case of Mrs. H., though equally striking, in its perfect semblance of inflammatory affections of different parts of the alimentary canal, was more limited in its sphere of diseased action, and less diversified in the nature of the attacks, only because fewer points of the spinal chain were implicated. Any one conversant in those complaints would infer that there existed acute tenderness of the upper cervical and upper lumbar, and perhaps of the two or three lower dorsal vertebræ.*

It is an extremely fortunate circumstance, although one

* Since the above was written this lady was again visited, in consequence of a violent spasmodic pain of the stomach, with which she was seized, followed by sickness, retching, and violent pain of back. She was relieved by hot fomentations to the spine, and aperients. She describes herself as subject to these spasmodic attacks ever since her long illness, that they always attack the stomach or abdomen first, and then, as she expresses it, "fly to the back," and fix there with increased violence. As there was now an opportunity of which could have been little anticipated, that the most trivial irritation of the minutest portion of the medullary column may, and almost always does, induce this corresponding tenderness. Indeed, it is a symptom so constantly present, and sometimes to so acute a degree, that it seems wonderful it should have been so long unobserved or undervalued. It appears to have completely escaped even so observant and refined a symptomatologist as Dr. Marshall Hall. The public owe much to Mr. Abernethy and Mr. Brodie, for having directed attention to some of those diseases of irritation, and for having distinguished them from the more serious affections of the bones. Such are the cases of hysterical pains in the spine, or hysterical tenderness, which the latter gentleman is said to make mention of in his lectures, and those extraordinary nervous affections pointed to in his excellent work on Diseases of the Joints, as bearing a striking resemblance to ulceration of the cartilages. Mr. Abernethy, in his essay on the Constitutional Origin of Local Diseases, has given several cases, which, it will be shown, were evidently those of simple irritation of the spine; but although, with his usual discrimination, he has distinguished them from the more formidable disease of the vertebræ, he seems not to have attached sufficient importance to the tenderness on pressure. He appears to have regarded it rather as one of the many anomalous symptoms connected with nervous irritation, and dependent on disorder of the digestive organs, than as the immediate source of the most distressing and greater number of them.

This symptom is, in fact, usually altogether overlooked in general practice. The patient unfortunately seldom complains of the back, and most frequently does not know that it is in the slightest degree affected. Hence, when the complaints are such as do not very obviously lead to an examination of the spine, the cough, pain of chest, oppressions, palpitations, the intense headachs, the spasmodic or apparently inflammatory affections of the abdomen, which fill the sufferer with such apprehension, present also the most obvious objects of treatment to the physician. It should never be forgotten that all affections at the sources of nervous power, or origins of nerves, are indicated by pain or disturbed action at the minute and distant extremities; and that this must hold true with respect to the brain ascertaining the truth of former conjectures, it was not neglected. There was extreme tenderness of all the lumbar vertebræ, and a slight degree at the seventh dorsal: she feels the pain in this last situation acutely when under the influence of the attack.

and spine, as well as with any of the great nervous trunks in which the phenomenon is more frequently observed. The following case of general irritation of the spine will serve to show how singularly true they sometimes are in the corresponding pains.

III. Bridget Leary, ætatis twenty-two, complains of constant distressing headachs, with oppression and shrill piping noise in breathing, pains in all her joints, pain at the pit of the stomach, in the sides, round the hips, in the limbs and feet. All these pains are increased by motion or exertion of any kind, and relieved by the recumbent position. She is weak, and troubled with palpitations; there is some feverishness, with whiteness of tongue, but the skin is cool; complains of continued sense of burning in the epigastrium, increased much by stooping or straightening herself, by over-bending or extending the spine; says "she often thinks her stomach will light;" feels as if her arm would break when she lifts it, she is seized with such pain there and in the axilla. The oppression is worse at night than in the day time.

On examining the spine, the whole column was found acutely tender; pressure at the first or second vertebra occasioned pain, which shot forward from the occiput to the brow; a little lower, pain was excited at the larynx; on pressing one of the lower cervical, it occurred at the point where the trachea dips behind the sternum; on pressing the upper dorsal, at the middle of the sternum; from the third or fourth dorsal to the eighth or ninth, it was excited at the ensiform cartilage; yet lower, at the sides, and in the lumbar vertebræ, pain was excited in the iliac and pubic regions. Pressure behind the trochanter produced pain at the crista of the ilium, at the inside of the thigh, and also in the sides, or in the opposite hip. On the thigh or knee, it excited pain in the shins and toes. The pain was more acute on pressing the first or second cervical, and seventh or eighth dorsal, than any others; which accounts for the headach and pain of stomach having been the most constant and distressing of all the symptoms.

This is by no means either one of the most uncommon or worst forms of spinal irritation. It may be of use, perhaps, to compare it with a case of chronic disease from injury, in which the analogy seems sufficiently strong to assist or influence our views of its nature.

IV. William Collins, a tall, spare man, aged fifty, about two years since, while working in a mill, had his coat seized, and was caught up by the wheel. The machinery was stopped in sufficient time to prevent his being ground to pieces, but his shoulders, back, and neck, were much crushed and injured. He was long ill, and had but an imperfect recovery, remaining affected with paralysis of the upper extremities, contraction of the fingers and debility of the lower limbs; he also suffered from pain and stiffness of the muscles of the neck, pains in all the limbs and joints, with crackling noise on motion, as in chronic rheumatism, and pains frequently in the head, chest, or abdomen. He has not latterly complained of the back.

The examination of the spine, which was universally tender, gave the following results:

Pressure on the first or second cervical vertebra occasioned pain over the brow; on the second or third, above and about the larynx; on the lower cervical, the lower part of the trachea as it enters the chest, and also at the top of the shoulder and in front of the chest. Pressure on the upper dorsal occasioned it at the superior part of the thorax; on the seventh or eighth, at the ensiform cartilage; on the tenth or twelfth, at the umbilicus. On the upper lumbar, at the sides and pubic region; on the lower lumbar and sacrum, at the groins, hips, and thighs. Behind the trochanter, at the knee and ankle.

When but one point of the cord is affected, the symptoms are proportionally simple: they are more apt to deceive the practitioner, from their resemblance to those chronic local diseases in which the constitution is little disturbed, and because, as has been already mentioned, the patient scarcely ever complains of the back. The following, in which pain of stomach, or at the ensiform cartilage, was the chief ailment, are among the very commonest of these cases.

V. Nelly Neville, ætatis twenty-two, complains of pain at the pit of the stomach, which commenced about two months since, and has continued without intermission. She has slight cough, with general languor and weakness; says she is much tormented with headach, and latterly with pain and stiffness at the back of the neck, which are sometimes relieved by tossing or throwing back the head. Her appetite is bad; tongue whitish; eyes slightly yellow. Cannot lift a can or basket, without bringing on or increasing the pain. There is tenderness at the seventh dorsal vertebra, pressure on which excites the pain at the stomach; no tenderness of the cervical. The catamenia are regular, but deficient.

This young woman was perfectly cured in a few days by a few purgatives, blistering over the affected part of the spine, and tonic bitters with acids.

VI. J. Enright, ætatis forty, complains of pain at the lower end of the sternum, with soreness of the part, and slight cough: has been ill with it now for two months; tongue whitish, bowels natural; general health tolerably good. There is extreme tenderness about the seventh or eighth dorsal vertebra, pressure on which occasions a darting pain from thence to the sternum, as if he was pierced by a sword.

Cured by purgatives, though more slowly than the foregoing patient. He was a labourer, and unwilling to blister, as it would oblige him to give up his work.

A case, similar in symptoms, though (if we may judge by its termination) widely differing in its nature, occurs in our notebook about the same period. Organic disease acts in the first instance very much like that strictly called functional, simply by pain, or by disturbance or interruption of function. This case, therefore, while it illustrates what Dr. Abercrombie has so strongly inculcated, the dangerous nature of affections of the spine originating in injury, points out the symptoms which must necessarily result from disturbed function of that particular part, and is tolerable evidence of their dependence on it in those lighter and less dangerous complaints.

VII. A middle-aged man, descending from a wall, fell backwards, and came against a rough stone, which occasioned a bruise about the seventh dorsal vertebra. He thought very little of the accident for some days, although there was slight pain and soreness, but then became affected with constant pain at the pit of the stomach; for relief of which he applied at the dispensary. Pressure on the tender vertebræ produced an instant pain at the pit of the stomach. Purgatives and fomentations to the spine were ordered, and he was desired to come on the next day of attendance, when, if not better, he was to be bled and blistered. Nothing, however, was heard of him for three weeks, when a report came that he had died suddenly. On inquiry, it was found he was so much relieved by the medicines that he did not think it necessary to attend: he, nevertheless, had an unaccountable languor and incapability of exertion about him, and occasionally kept his bed. On the last morning of his life, he said he felt pretty well, and would get up and go to work; but, while dressing with this intention, suddenly fell back and expired.

It is not meant, of course, to assume that spinal tenderness is never a mere symptomatic affection: we shall have frequent occasion to speak of cases which were evidently produced by, and dependent on, intestinal, dental, or other irritations. Mr. Abernethy cites some, which, with every appearance of reason, he attributes to disease of the digestive organs; but, even in many complaints of this kind, especially when existing for any length of time, the spinal affection becomes a serious and absolute disease, reacting on and increasing the disorder which gave it existence, or producing a new train of symptoms proper to itself.

In our early inquiries it was so frequently met with, especially in females, that we found it necessary to make a general examination of the patients attending the dispensary, to ascertain in how far it was to be regarded as an independent affection. The result showed that it was very seldom wanting where those nervous symptoms supposed to indicate disorder of the spinal cord were present, and where there was no local disease to which it could be attributed; while, in almost every instance in which acute or chronic local disease was found to exist, no such tenderness could be detected. Those few cases of local disease in which it was observed, were chiefly acute affections of the liver, or inflammatory complaints resulting from injury; and the former of these were, it is probable, rather an effect than a cause: at least, it is, in any other view, extremely difficult to account for its absence in some intense inflammations which are met with.

The minute attention to the spine which these examinations induced, led to much more interesting inferences than could have been at all anticipated. The great tenderness of the cervical vertebræ, in some cases of sudden fits of insensibility, suggested its existence in epilepsy,* in some forms of which it was found invariably present; a fact very well agreeing with M. Esquirol's dissections in this disease, which so frequently displayed morbid changes in the cord or its membranes. The connexion observable between tenderness at the same part and headach, soreness and pain of stomach, in cases of spinal irritation, occasioned the discovery of its existence in continued fever, in all cases where there was much disturbance of the stomach and head, and induced a suspicion that it was equally the source of Dr. Clutterbuck's cerebral inflammation and M. Broussais'

* When speaking of the diseases induced by irritation of the cervical portion of the cord, we shall give some most extraordinary cases of cure of this dreadful malady.

gastro-enterite. The occasional occurrence of shivering fits in spinal cases pointed out some analogy between them and intermittents, and, as was conjectured, most acute tenderness of the whole spine was ascertained to exist in the very few of these complaints which fell within our observation during the last month. It was also detected in numerous cases of neuralgia, in many of paralysis, and in all that class of complaints called mimosa which came under our notice. In short, we were finally driven to the conclusion that the greater number of these disorders either wholly depend on some affection of the spinal column, or are strangely and importantly connected with it. It must, of course, be obvious that, in cases usually terminating favorably after a longer or shorter period, morbid anatomy can afford no satisfactory evidence in support of such an opinion. It can admit of little more than hypothetical or analogical proof; but if it be acknowledged that the symptoms of these complaints are such as, from the functions of the spinal cord and nerves, we know any derangement of them may occasion; if it be shown that mechanical injuries of its individual portions occasion symptoms which correspond with those induced by irritation of the same portions; that injuries producing a more general affection induce the precise symptoms which we attribute to general spinal irritation; and, above all, that those affections supposed to depend on this irritation are often readily cured by its removal, after having resisted every other plan of treatment; we shall have attained a degree of probability quite as great as we are accustomed to depend on, or give our assent to, in the more established points of medical reasoning.

It is, after all, satisfactory to reflect that, if more extensive experience should seem to declare the foregoing inferences untenable, the facts must nevertheless hold their ground, and furnish useful matter to the symptomatologist. We have already said enough to show that one of the most important desiderata in diagnosis, is that which would enable us to distinguish between the diseases of irritation and inflammation, between those of function and of structure, between those attacking the sources of nervous influence, yet developing themselves only in distant tissues or organs, and the simpler affections of those organs dependent upon local causes. If any new illustration of this were wanting, we have it in the late Croononian lecture by Dr. Hawkins on affections of the brain, in which he cites many instances where, from the ambiguity of the symptoms, the disease was supposed to depend on nervous sympathy, and, from the consequent remissness of treatment, terminated in fatal apoplexy. If our views be correct, spinal tenderness would have been discovered in those cases attributed to nervous sympathy, but would have been absent in the inflammatory and organic. This assertion must be received at present as a general one, and with some qualification, especially in affections of the base of the brain immediately adjoining the medulla oblongata.

As, in the present state of our knowledge, strict distinctions, founded on the supposed nature of various spinal affections, must be liable to much error, it seems proper to offer such only as the symptoms would obviously indicate, without assuming that they are in all instances founded on any specific difference in the nature of the complaint. The following may be said to include all which have fallen within our experience.

1st. Cases of irritation of the spinal cord, with tenderness at one or more points of the spine.

2d. Cases with symptoms resembling the foregoing, but unattended by spinal tenderness.

3d. Cases of obscure spinal disease, in which there is extreme pain on twisting or bending the spine, or on lifting weights, but no tenderness on pressure.

4th. Cases of acute spinal inflammation, attended by pains of a rheumatic character, and by many of the symptoms of general irritation of the cord; but chiefly marked by high fever, excruciating pain, and tenderness in some part of the back, occurring in paroxysms on the slightest motion, and often occasioning or ending in paralysis.

5th. Cases of chronic spinal inflammation, attended by pains resembling those of chronic rheumatism, with many of the symptoms of general irritation of the spine, and the usual tenderness on pressure.

6th. Cases of caries of the vertebral bones and distortion, which have been so ably treated of by many eminent writers, it is merely necessary to name, as much rarer diseases than any of the foregoing, but having very many symptoms in common with them, and affording frequent grounds for apprehension and error, when the diagnosis is not attentively studied.

7th. The same may be said of those organic diseases of the spinal cord whose pathology Dr. Abercrombie has taken such pains to illustrate. We have met with very few of them in the course of our practice, and those were such as offered little new or interesting on the subject.

1. *Of Irritation of the Spinal Cord.*

Before entering more particularly into this subject, it may be necessary to make some observations on the use of the word irritation, which has already been of such frequent occurrence. It is, perhaps, one of those phrases very difficult of definition, which, in an obscure science, we are rather constrained than pleased to use, in speaking of the causes of phenomena beyond our explanation. We mean simply to express by it any stimulus acting on the whole or parts of the system, through the sensorium, without primary vascular excitement; and it would appear very probable, even in those diseases apparently originating in vascular excitement, as in inflammations, that the increased action of the vessels is not the first step in the process, but that a certain action or impression, as in the diseases of simple irritation, previously takes place in the sensorium. It does not seem easy to imagine that the actions of any vital part can take place independently of the centre and source of all vital phenomena, any more than sensation or motion could; and it might be a question, if the communication by ganglionic nerves to an injured part could be cut off as perfectly as by the spinal, whether local inflammation could take place at all?

Whatever the nature of nervous irritation may be, we believe its existence is unavoidably admitted by the profession;* and, though so little comprehensible, a knowledge of its character and action is of vast practical importance in medicine, forming the great distinctive marks between the most opposite classes of diseases. We can observe all its possible effects, though we cannot detect the mode, and note the anomalous discrepancy by which to distinguish its symptoms from signs of diseased structure, when we cannot offer any diagnostic of which we could give the rationale. "Ought it not to be a question," inquires Mr. C. Bell,

* Mr. John Bell's opinion, that all nervous disorders depend on the circulation of blood in the brain, was founded on the idea that, as the brain was insensible, there could be no such thing as nervous irritation. But he was not then aware that impressions might be received, and distinct actions or sympathies excited, through the medium, not of the brain, but of distinct portions of the spinal cord, now the acknowledged source of all sensation and all motion; and that in this way the functions of the brain, as well as of every other organ in the body, might be either interrupted or rendered more energetic, without its being the seat or medium of the irritation. The fact stated by Desmoulin and Magendie, in their late work on the Vertebrata, that feeling and thought are not only distinct and independent faculties, but are distant from one another in their seats, seems likely to lead to some very interesting speculations on this subject.

"what nervous affections are consequent on trivial irritation?" Perhaps, there is not another connected with medical science, of such real importance, that has been so much the subject of loose and doubtful opinion. Facts, familiar to every one, go far to show what we hope subsequent cases may more fully establish, that there is no manner in which the functions of the senses, sensation, motion, circulation, secretion, may be excited, diminished, or extinguished by organic disease, that they may not also, even more suddenly, by nervous irritation. The heat of the sun on the bare head, the abstraction of blood, the motion of a worm in the stomach, will excite convulsions as effectively as inflammation or altered structure of the brain or spine. The sight of a disgusting insect induces vomiting as certainly as carcinoma of the stomach; the lancing of a whitlow, syncope, as truly as ossification of the coronary arteries of the heart. There is, in short, no affection of sense or motion so painful or paralysing, that mere irritation may not occasion; and it becomes a doubt only how trivial a degree may produce the worst of these effects, or whether the morbid action bears any regular proportion to the intensity of irritation.

Though the relations of many parts of the system are still so mysterious, and the sympathies so complicate and extraordinary, as to defy all explanation by nervous communication, much may in this way be understood, especially of the spinal cord and its connexions. Its physiology has been so very much elucidated by late experiments, that a little consideration would suggest to us the complaints likely to be produced by affections of any individual portion of it. From its continuity with the brain (that part to which intellectual actions have been assigned), and their known reciprocal sympathies, we should expect its diseases would excite many symptomatic disorders of that organ, as pain, vertigo, delirium, &c., which we shall find not uncommon. As the origin of all sensation and motion, we might anticipate simply painful, or spasmodic, or paralytic affections in any part of the body, or that general loss of feeling and motion in which we sometimes see persons lie, inanimate and powerless; the body still, the eyes fixed, the functions of respiration carried on imperceptibly, yet fully sensible of all that is going on around them. As it includes the origin of the fifth pair, which is found to be essentially necessary to every organ of sense, except sight, in the exercise of its functions, and even to sight to be accessory, irritation or disease near the trunks of that nerve should induce disturbance of the functions of the senses, or temporary paralysis of all or any of them, or painful affections of the extremities of these nerves themselves, as in the orbital, or facial, or alveolar branches. As the seat of the respiratory functions, we should be inclined to attribute to its disorder many complaints of the respiratory system. Near to the origin of the fifth are the roots of the respiratory nerves, the glosso-pharyngeal, the eighth pair, the spinal accessory, the phrenic; derangement or irritation of which should occasion affections of the throat, respiratory muscles, lungs, diaphragm, and stomach, loss or change of voice, hoarseness; crowing, croupy, or wheezing respiration; barking cough, globus hystericus, spasms of the chest or stomach, difficult deglutition, hiccough, weeping, crying, laughing, &c. Irritation at the root of the cervical nerves might induce, by communication, any of the foregoing symptoms, or occasion pain, stiffness, rigidity, or spasm of the muscles of the neck or arm, or numbness or paralysis. To irritation at the origin of the dorsal nerves we might attribute oppression, palpitation, pain in the anterior of the chest or stomach, or sides; and, at the origin of the lumbar nerves, abdominal tenderness, colic, constipation, pains in the loins, hips, extremities, with injury or paralysis of the bladder, or of the lower limbs.

Lastly, the whole of the medulla spinalis, including the origin of the eighth pair, goes to form the ganglionic system of nerves. These supply all the muscles of involuntary motion, and all the viscera; and we have reason to suppose are mainly concerned in the secreting processes, among which Dr. Wilson Philip places the disengagement of caloric. They are distributed in networks round the arteries, perhaps solely supplying them with nervous power, and rendering them like the heart, though independent of, yet liable to be influenced by, any considerable portion of the brain or spinal marrow. In irritations of the whole or a great portion of it, therefore, we should anticipate irregular action of the heart, evinced in palpitations or in approaches to a suspension of all action, and syncope; interruption of the secretions, evinced in the oppressed breathing, failure of appetite, and flushings or burning heats, or universal shiverings, or coldness of the extremities or of particular members; and irregularities of the circulation, evinced in local, and often violent, determinations, or in loss of tone and vascular debility. How accurately these conjectures are borne out by subsequent cases, the reader will have an opportunity of considering.

[To be continued.] 93.

